# The prognostic value of microvascular invasion in early-intermediate stage hepatocelluar carcinoma: a propensity score matching analysis

**DOI:** 10.1186/s12885-018-4196-x

**Published:** 2018-03-12

**Authors:** Junyi Shen, Jun Wen, Chuan Li, Tianfu Wen, Lvnan Yan, Bo Li, Jiayin Yang, Changli Lu

**Affiliations:** 1 Department of Liver Surgery and Liver Transplantation Center, West China Hospital, Sichuan University, Chengdu, Sichuan Province 610041 China; 2 Department of pathology, West China Hospital, Sichuan University, Chengdu, China

**Keywords:** Microvascular invasion, Prognosis, Hepatocelluar carcinoma, Propensity score matching, Barcelona clinical liver Cancer

## Abstract

**Background:**

Microvascular invasion (MVI) is well established as a negative prognostic factor for hepatocelluar carcinoma (HCC). However, its prognostic value in different subgroups of Barcelona Clinical Liver Cancer (BCLC) stages remains to be elucidated.

**Methods:**

Four hundred fifty-eight MVI-negative and 204 MVI-positive patients who underwent hepatectomy were retrospectively analyzed. After propensity score matching (PSM) analysis, 187 pairs of matched patients were generated. Long-term survival was compared by the Kaplan–Meier method.

**Results:**

Patients with MVI commonly had more advanced tumors. All the patients with MVI had significantly worse survival rate compared to the patients without MVI before and after PSM(*p* < 0.001). In the subgroup analysis, BCLC stage A HCC patients without MVI had better prognosis than those with MVI before and after PSM (*p* < 0.001 and *p* = 0.024). For BCLC stage B HCCs, long-term survival was significantly better for patients without MVI before PSM(*p* = 0.001). However, the overall survival (OS) rate was comparable between both groups after PSM (*p* = 0.682), although MVI-positive group had a higher rate of recurrence (*p* = 0.011).. Surgery type, satellite lesions, tumor size, and serum ALT level were statistically significant factors associated with survival in MVI-positive group. Tumor number, tumor size and neutrophil to lymphocyte ratio (NLR) were predictors of survival in MVI-negative group.

**Conclusions:**

Its prognostic value in different subgroups of BCLC stages differed. MVI is an independent predictor of prognosis in patients with BCLC stage A. For BCLC stage B HCCs, MVI-positive group had poor prognosis through more advanced HCCs.

## Background

Hepatocellular carcinoma (HCC) is one of the most common causes for cancer death worldwide. [1] Barcelona Clinic Liver Cancer (BCLC) staging system is widely accepted because it links the tumor stage to treatments indication and it has good distinctive power for prognosis. [2] Surgical resection (SR) is currently the main modality of curative treatment for patients with good functional liver reserves. The BCLC staging system and the American Association for the Study of Liver Diseases (AASLD) do not recommend surgical resection for HCC beyond the Milan criteria (single tumor ≤5 cm or up to three nodules, each ≤3 cm in size, and without major vascular invasion), [3] however, some western and eastern centers have reported that hepatectomy could bring survival benefits for patients with HCC beyond the Milan criteria with well-preserved liver function compared to transcatheter arterial chemoembolization (TACE) [4, 5]. Additionally, surgical resection allows pathology inspection, providing sufficient pathological information, such as vascular invasion and satellite lesions. HCCs are characterized by high presence of invasion of portal vein resulting in intrahepatic spread through macro or micro tumor emboli. Vascular invasion has been widely reported to be a poor prognostic factor for HCC for both hepatectomy and liver transplantation [6–9]. In the BCLC and AALSD staging systems, HCC with macrovascular invasion is classified as advanced stage tumors. Currently, the presence of MVI could be detected in early-advanced stage HCC with well liver reserve function [10], however, the prognostic importance of MVI is not defined in current staging systems for HCCs. For intermediate-advanced HCCs, other risk factors, such as satellite lesions, heavy tumor burden and major vascular invasion, occurred. The impacts of microvascular invasion on intermediated-advanced stage HCC’ prognosis is not clear. One study suggested that MVI did not impact overall survival in patients with very early HCC (≤2 cm) and it only provides useful information on progress for HCCs > 2 cm [10]. This might suggest that the role of MVI in different stage HCCs varies.

MVI is not easily detected preoperatively, many investigators sought out to clarify the predictors of MVI. AFP > 400 ng/ml, large tumor size (> 8 cm), tumor number (> 3), and Protein Induced by Vitamin K Absence II (PIVKA-II) level (> = 200 mAU/ml) has been validated as risk factors associated with the presence of MVI [11, 12]. Moreover, with advances in imaging technology, specific imaging findings and imaging methods can provide useful information to find out the presence of MVI [13, 14]. All these methods can be helpful in clarifying the role of MVI in different subgroups of HCCs.

In this study, we classified all patients as MVI-positive group and MVI-negative group. In order to reduce the selection bias and eliminate the confounding factors, propensity score matching (PSM) was used to investigate the role of MVI on HCC after surgery. This study aims to investigate (1) the prognostic value of MVI in HCC before and after PSM; (2) the influence of MVI on the prognosis of patients undergoing resection for BCLC stages A/B HCCs; (3) the discrepancy in risk factors associated with prognosis between MVI-positive group and MVI-negative group.

## Methods

### Participants

Six hundred sixty-two patients with HCC who underwent hepatectomy from April 2007 to October 2014 were included from our prospectively maintained database in Department of Liver Surgery & Liver Transplantation Center in West China Hospital. The included criteria are as follows: (1) patients did not receive radiofrequency ablation preoperatively; (2) Child-Pugh A/B; (3) without concurrent cancers. The excluded criteria are as follows: (1) recurrent HCC (2) Surgery-related death within 30 days after surgery; (3) extrahepatic metastasis; (4) major vascular invasion; (5) positive surgical margin; (6) incomplete follow-up data. The preoperative diagnosis of HCC was based on either two typical imaging findings or a combination of AFP > 400 ng/ml and one imaging finding (liver ultrasound or computed tomography (CT) or magnetic resonance imaging (MRI)). HCCs were histologically confirmed by experienced liver pathologists in West China Hospital. Clinical variables including gender and age and pathological characteristics including liver cirrhosis evaluated by Ishak score, tumor size, tumor number, the degree of tumor differentiation, satellite lesions, MVI and major vascular invasion were collected from pathological reports. Routine blood tests (Platelet count/lymphocyte count/ neutrophil count. etc), alanine aminotransferase (ALT), aspartate aminotransferase (AST), total bilirubin (TBIL), serum albumin (ALB), serum alpha-fetoprotein (AFP), status of hepatitis B viral (HBV) infection, HBV-DNA level was measured 2 days before surgery. Surgery type was considered minor liver resection if one segment was removed; major liver resection if two or three were removed; or extended liver resection if more liver segments were removed. This study was approved by the ethic committee of West China Hospital, and written informed consent forms were obtained from all the participants.

### Definitions

In the current study, BCLC A: single tumor or 3 nodules ≤3 cm. BCLC B: 2–3 lesions, with at least 1 lesion more than 3 cm in diameter or more than 3 lesions of any diameter [15]. Positive HBV-DNA is defined as HBV-DNA load more than 10^3 copies/ml. Surgery type included minor liver resection which involved one liver segment, major liver resection which involved 2–3 liver segments, and extended liver resection which involved more than 3 liver segments, such as extended left hemihepatectomy or right hemihepatectomy. NLR is defined as the neutrophil counts divided by the lymphocyte counts. PLR is defined as the platelet counts (PLT) divided by the lymphocyte counts. MVI is defined as microscopic tumor emboli within within the central hepatic vein, the portal vein, or the large capsular vessels [10, 16]. Satellite lesions were defined as tumors ≤2 cm in size that were located within a distance of 2 cm from the main tumor [17].

### Follow up

All patients were regularly followed up at the first, third and sixth months in the first half year after the operation, every 3 months throughout the subsequent 3 years, and every 6 months thereafter as described in our previous study [18]. Routine blood tests, AFP levels, liver function tests, HBV markers and HBV-DNA levels, and liver ultrasound were included at each follow-up examination. Recurrent lesions were confirmed by CT or/and MRI, or by biopsy. If there was HCC recurrence, Patients were evaluated by multidisciplinary team (MDT) in West China Hospital for treatment guidance based on the status of tumor and general condition. Salvage liver transplantation, resection, ablation, TACE and palliative therapy was recommended based on the status and general condition of the recurrent tumor. Patients were administrated anti-viral therapy, such as Entecavir (0.5 mg/day), if their HBV-DNA levels were > 1.00E + 03copies/ml before, after surgery and during follow up. The recurrence free survival (RFS) time was defined as the interval between resection and the first confirmed recurrence. The overall survival (OS) time was defined as the time from the date of surgery to the time of death, or last follow-up if death had not occurred. The median postoperative follow-up period was 34.6 months (range, 0–120 months).

### Statistical analysis

The statistical analysis was performed using SPSS 20.0 software. Continuous variables were described by medians + standard deviation (M + SD) and were assessed by the Student t test. Categorical variables were presented by frequencies and were compared by the chi-square test and fisher exact test, respectively. Cumulative RFS rate and OS after hepatectomy was calculated by the Kaplan–Meier method, and group differences were compared using the log–rank test. Cox proportional hazards models with forward stepwise were used to assess risk factors to predict the prognosis of HCC after hepatectomy. Significant variables in the univariate analysis were included in the multivariate analysis. Two-tailed *P*-values of < 0.05 were considered statistically significant.

Variables with statistically significant differences between groups might result in misleading outcomes. Potential covariables included in PSM were age, gender, the status of HBV infection, AFP, AST, ALT, PLR, NLR, differentiation, satellite lesions, Ishak score tumor number, tumor size, surgery type and blood transfusion. Propensity scores were estimated using a logistic regression model. A one-to-one nearest neighbor matching algorithm was applied with a caliper of 0.2 and without replacement. Standardized mean differences and linear plot of individual differences were shown in order to examine the outcome of PSM. One hundred eighty-seven matched pairs were generated and applied in further analyses.

## Results

### Patient’s baseline characteristics before and after PSM analysis

Characteristics of MVI-positive and MVI-negative patients are summarized in Table 1. Of all the patients, 204 patients (30.8%) had MVI and 458(69.2%) had no. The incidence of positive HBsAg, positive HBV-DNA load, serum AST level, the rate of NLR (≥3) and PLR(≥111) were lower in MVI-negative group. MVI-positive group had more male patients and less patients of age > 60y. And MVI-positive group had poor tumor status including poorer tumor differentiation, higher proportion of satellite lesions, multiple HCCs, higher rate of patient with AFP greater than 400 ng/ml, and larger tumor size. Patients without MVI had higher rate of minor liver resection and less blood transfusion while patients with MVI were more likely to have extended liver resection and more blood transfusion. Based on BCLC classification, MVI-positive patients had more advanced stage HCC. There were no statistically significant differences in Ishak score, serum ALT level, ALB level and TBIL level.

**Table 1:** Comparison of baseline demographics of patients with or without MVI

		MVI(+)	MVI(−)	*p* value
		*n* = 204	*n* = 458	
age > 60y		37(18.1)	135(29.5)	0.002
Gender(male/female)		19/185	72/386	0.028
Positive HBsAg		186(91.2)	390(85.2)	0.034
Positive HBV-DNA load(>10^3 IU/ml)		97(47.5)	203(44.3)	0.448
AFP(> 400 ng/ml)		110(53.9)	179(39.1))	< 0.001
Differentiation	poor	109(53.4)	166(36.2)	< 0.001
	well-moderate	95(46.6)	292(63.8)	
Satellite lesion		49(24.0)	37(8.1)	< 0.001
Tumor number^**^	one	140(68.6)	367(80.1)	< 0.001
	two	27(13.2)	62(13.5)	
	more	37(18.1)	29(6.3)	
Tumor size(cm)		7.9 ± 4.0	6.0 ± 3.3	< 0.001^*^
Ishak score	5–6	139(68.1)	285(62.2)	0.161
	0–4	65(31.9)	173(37.8)	
BCLC classification^**^	A	144(70.6))	373(81.4)	0.002
	B	60(29.4)	85(18.6)	
Lg10ALT(U/l))		1.7 ± 0.3	1.6 ± 0.3	0.457
Lg10AST(U/l)		1.7 ± 0.2	1.6 ± 0.3	0.007
ALB(g/l)		41.4 ± 4.2	41.5 ± 4.4	0.928
TBIL(mmol/l)		15.1 ± 7.2	15.1 ± 6.9	0.938
PLR	≥111	87(42.6)	146(31.9)	0.008
	< 111	117(57.4)	312(68.1)	
NLR	≥3	76(37.3)	118(25.8)	0.003
	< 3	128(62.7)	340(74.2)	
Surgery type	extend	44(21.6)	95(20.7)	0.006
	major	81(39.7)	130(28.4)	
	minor	79(38.7)	233(50.9)	
Blood transfusion		22(10.8)	28(6.1)	0.039
Recurrence site	intra-hepatic	142(69.6)	238(52.0)	< 0.001
	extra-hepatic	26(12.7)	43(9.4)	
Recurrence treatments	salvage LT	1(0.5)	7(1.5)	< 0.001
	re-resection	15(7.4)	33(7.2)	0.066
	radiofrequency ablation	9(4.4)	31(6.8)	< 0.001
	TACE	72(35.3)	93(20.3)	< 0.001
	pallitive therapy	64(31.4)	97(21.2)	< 0.001

^*^indicates statistically significant, ^**^compared by the Mann–Whitney rank sum test

*MVI* microvascular invasion, *HBsAg* hepatitis B virus surface antigen, HBV-DNA hepatitis B viral DNA, *AFP* alpha-fetoprotein, *BCLC* Barcelona Clinic Liver Cancer, *ALT* alanine aminotransferase, *AST* aspartate aminotransferase, *PLT* Platelet, *ALB* albumin, *TBIL* total bilirubin, *PLR* platelet to lymphocyte ratio, *NLR* neutrophil to lymphocyte ratio, *LT* liver transplantation, *TACE* transarterial chemoembolization

**Fig. 1:**
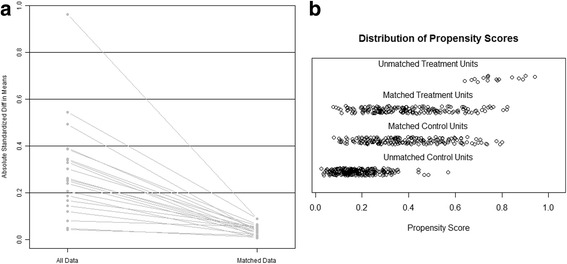
Evaluation of baseline characteristics after PSM. **a** line plot of standardized differences before and after matching. **b** dotplot of distribution of propensity score in either matched or unmatched groups. Graph was produced using routines from the MatchIt package. PSM: propensity score matching

Variables such as age, gender, status of HBV infection, AFP, AST, ALT, PLR, NLR, tumor differentiation, satellite lesions, Ishak score tumor number, tumor size, surgery type and blood transfusion were included in PSM analysis. One hundred eighty-seven pairs were generated, and no significant difference was observed between the MVI-positive and MVI-negative groups. One hundred thirty-eight patients (73.8%) and 49(26.3%) were classified as BCLC stage A and B for MVI-positive group, and 140 patients (74.9%) and 47(25.2%) were classified as BCLC stage A and B in MVI-negative group, respectively (Table 2). After PSM, the absolute standardized Difference means decreased to below 0.2. The propensity score in matched groups was evenly distributed (Fig. 1).

**Table 2:** Baseline demographics between patients with MVI and without MVI after propensity score matching(PSM)

		MVI(+)	MVI(−)	*p* value
		*n* = 187	*n* = 187	
Age > 60y		35(18.7)	36(19.3)	1.000
Gender(male/female)		169/18	168/19	1.000
Positive HBsAg		171(91.4)	171(91.4)	1.000
Positive HBV-DNA load(>10^3 IU/ml)		92(49.2)	92(49.2)	1.000
AFP(> 400 ng/ml)		98(52.4)	94(50.3)	0.756
Differentiation	poor	97(51.9)	95(50.8)	0.918
	well-moderate	90(48.1)	92(49.2)	
Satellite lesion		37(19.8)	31(16.6)	0.503
Tumor number^**^	one	134(71.7)	134(71.7)	0.620
	two	27(14.4)	32(17.1)	
	more	26(13.9)	21(11.2)	
Tumor size(cm)		7.5 ± 3.7	7.4 ± 3.9	0.924
Ishak score	5–6	126(67.4)	128(68.4)	0.912
	0–4	61(32.6)	59(31.6)	
BCLC classification^**^	A	138(73.8)	140(74.9)	0.906
	B	49(26.2)	47(25.2)	
Lg10ALT(U/l))		1.6 ± 0.3	1.6 ± 0.3	0.924
Lg10AST(U/l)		1.7 ± 0.2	1.7 ± 0.2	0.871
ALB(g/l)		41.5 ± 4.2	41.6 ± 5.0	0.810
TBIL(mmol/l)		15.0 ± 6.5	14.5 ± 6.9	0.460
PLR	≥111	77(41.2)	75(40.1)	0.916
	< 111	112(59.9)	110(58.8)	
NLR	≥3	62(33.2)	64(34.2)	0.913
	< 3	125(66.8)	123(65.8)	
Surgery type	extend	38(20.3)	46(24.6)	0.858
	major	74(39.6)	61(32.6)	
	minor	75(40.1)	80(42.8)	
Blood transfusion		18(9.6)	16(8.6)	0.858

^**^compared by the Mann–Whitney rank sum test

Abbreviation as Table 1

### Recurrence and follow up treatment

Tumor recurrence was seen 168 patients (82.3%) in MVI-positive group and 281(61.4%) in MVI-negative group (*P* < 0.001). Intrahepatic recurrence in MVI-positive patients occurred in 142 patients (69.6%), and 238 MVI-negative patients (52.0%) had intrahepatic recurrence. The proportion of intrahepatic and extraheptic recurrence in MVI-positive patients after hepatectomy was higher than that in MVI-negative patients.

If there was HCC recurrence, treatments included salvage transplantation, re-resection, radiofrequency ablation (RFA), TACE and palliative therapy. The most common follow up treatments were TACE and palliative therapy. The proportion of radiofrequency ablation and salvage LT was higher in MVI-positive group, while the proportion of TACE and palliative therapy was higher in MVI-negative group. The proportion of re-resection between both groups were not significantly different (Table 1).

### Survival analysis before and after propensity score matching (PSM)

**Fig. 2:**
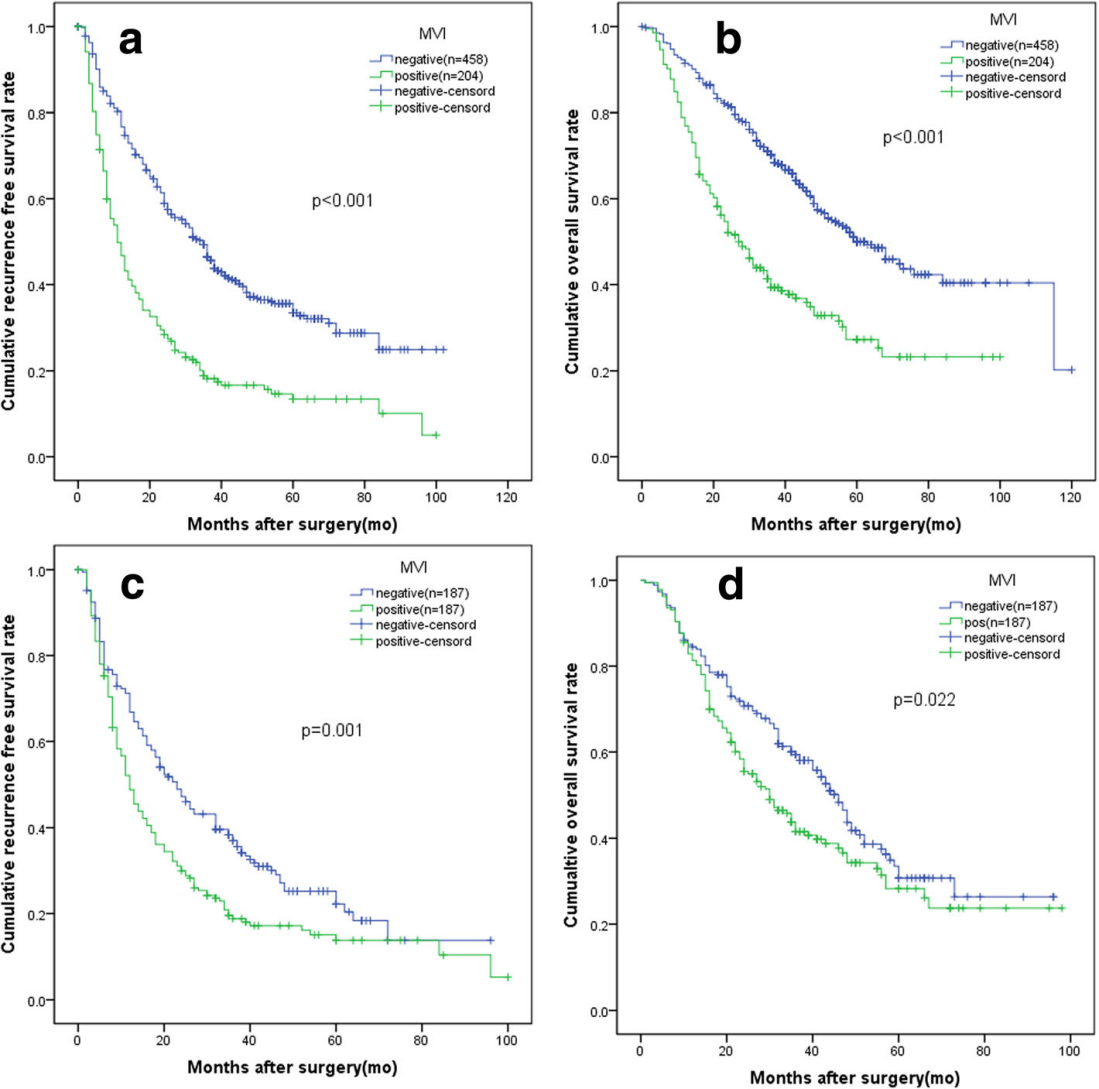
Comparison of patients with MVI and patient without MVI. **a** the recurrence free survival (RFS) rates before PSM (*P* < 0.001). **b** The overall survival rates (OS) before PSM (*P* < 0.001); **c** the RFS rates after PSM (*P* < 0.001). **d** The OS rates after PSM (*P* = 0.022) MVI: microvscular invasion PSM: propensity score matching

Comparison of RFS and OS between the two groups before and after PSM is illustrated in Fig. 2. Before PSM, the median RFS was 35 and 11 months for MVI-negative patients and MVI-positive patients, respectively. The 1-, 3-, and 5-year RFS rates were46.7, 18.2, and − 13.4% in MVI-positive group; and76.7, 46.5 and 33.6% in MVI-negative group, respectively (*P* < 0.001). The median OS was 60 and 27 months for MVI-negative patients and MVI-positive patients, respectively. The 1-, 3-, and 5-year OS rates were 77.0, 39.3 and 27.3% in MVI-positive group; and 91.4, 70.3 and 50% in MVI-negative group, respectively(*p* < 0.001).

After PSM, the median RFS was 23 and 12 months for MVI-negative patients and MVI-positive patients, respectively. The 1-, 3-, and 5-year RFS rates were48.8, 18.8, and 13.8% in MVI-positive group; and66.8, 37.0 and 22.2% in MVI-negative group, respectively (*P* = 0.001). The median OS was 46 and 30 months for MVI-negative patients and MVI-positive patients, respectively. The 1-, 3-, and 5-year OS rates were81.3, 41.5, and 26.1% in MVI-positive group; and 83.9, 59.4 and 30.7% in MVI-negative group, respectively(*p* = 0.022).

Multivariate Cox regression analysis by using forward stepwise yielded prognostic factors for survival (Table 3). Surgery type (extend liver resection vs. minor liver resection: hazard ratio(HR) 1.728, 95% confidence interval(CI):1.066–1.728, *p* = 0.027), satellite lesions(HR 2.428, 95% CI: 1.663–3.544, *p* < 0.001), tumor size (HR 1.108, 95% CI: 1.059–1.159, *p* < 0.001), and serum ALT level (HR 1.007, 95% CI: 1.004–1.010, *p* < 0.001) were all statistically significant independent factors associated with survival in MVI-positive group. Tumor number (HR 1.595, 95% CI: 1.292–1.969, *p* < 0.001), tumor size(HR 1.159, 95% CI: 1.117–1.202, *p* < 0.001) and NLR(HR 1.442, 95% CI: 1.056–1.971, *p* = 0.021) were predictors of survival in MVI-negative group.

**Table 3:** Independent prognostic predictors in all patients stratified by MVI in the multivariate Cox proportional hazards model

Multivariate analysis	MVI-positive (*n* = 204)		
Variables	HR	95%CI	*P* value
Sugery type			0.034
Major vs. minor			0.403
Extend vs. minor	1.728	1.066–1.728	0.027
Satellite lesions	2.428	1.663–3.544	< 0.001
Tumor size	1.108	1.059–1.159	< 0.001
ALT	1.007	1.004–1.010	< 0.001
Variables	MVI-negative(*n* = 458)		
Tumor number	1.595	1.292–1.969	< 0.001
Tumor size	1.159	1.117–1.202	< 0.001
NLR	1.442	1.056–1.971	0.021

*MVI* microvasuclar invasion, *HR* hazard ratio, *CI* confidence interval, *ALT* alanine aminotransferase

### Subgroup analysis based on BCLC stage

**Fig. 3:**
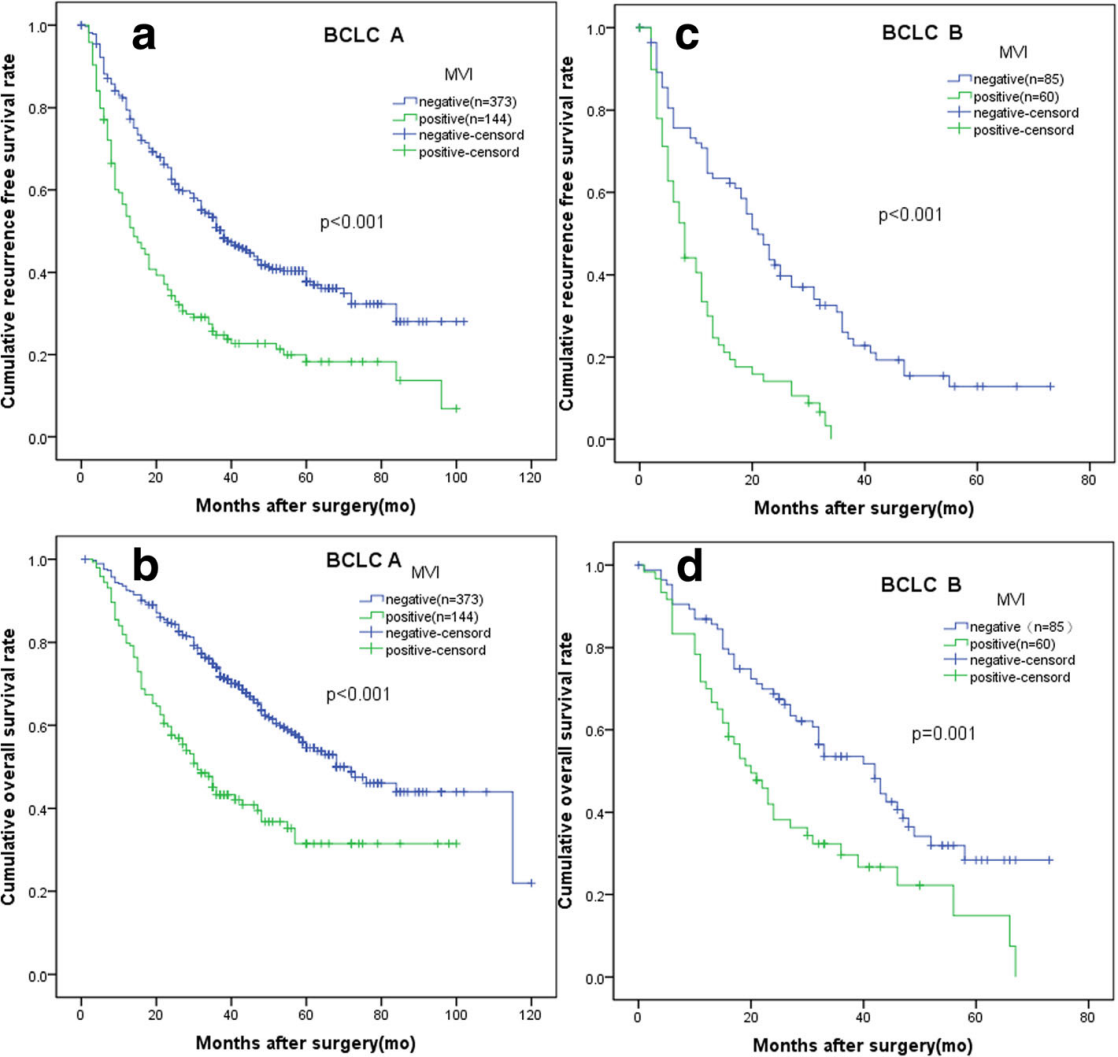
Comparison of patients with or without MVI stratified by BCLC classification before PSM. **a** the recurrence free survival (RFS) rates in patients with BCLC stage A HCCs (*P* < 0.001). **b** The overall survival (OS) rates in patients with BCLC stage A HCCs (*P* < 0.001). **c** the RFS rates in patients with BCLC stage B HCCs (*P* < 0.001). **d** The OS rates in patients with BCLC stage B HCCs (*P* = 0.001)

**Fig. 4:**
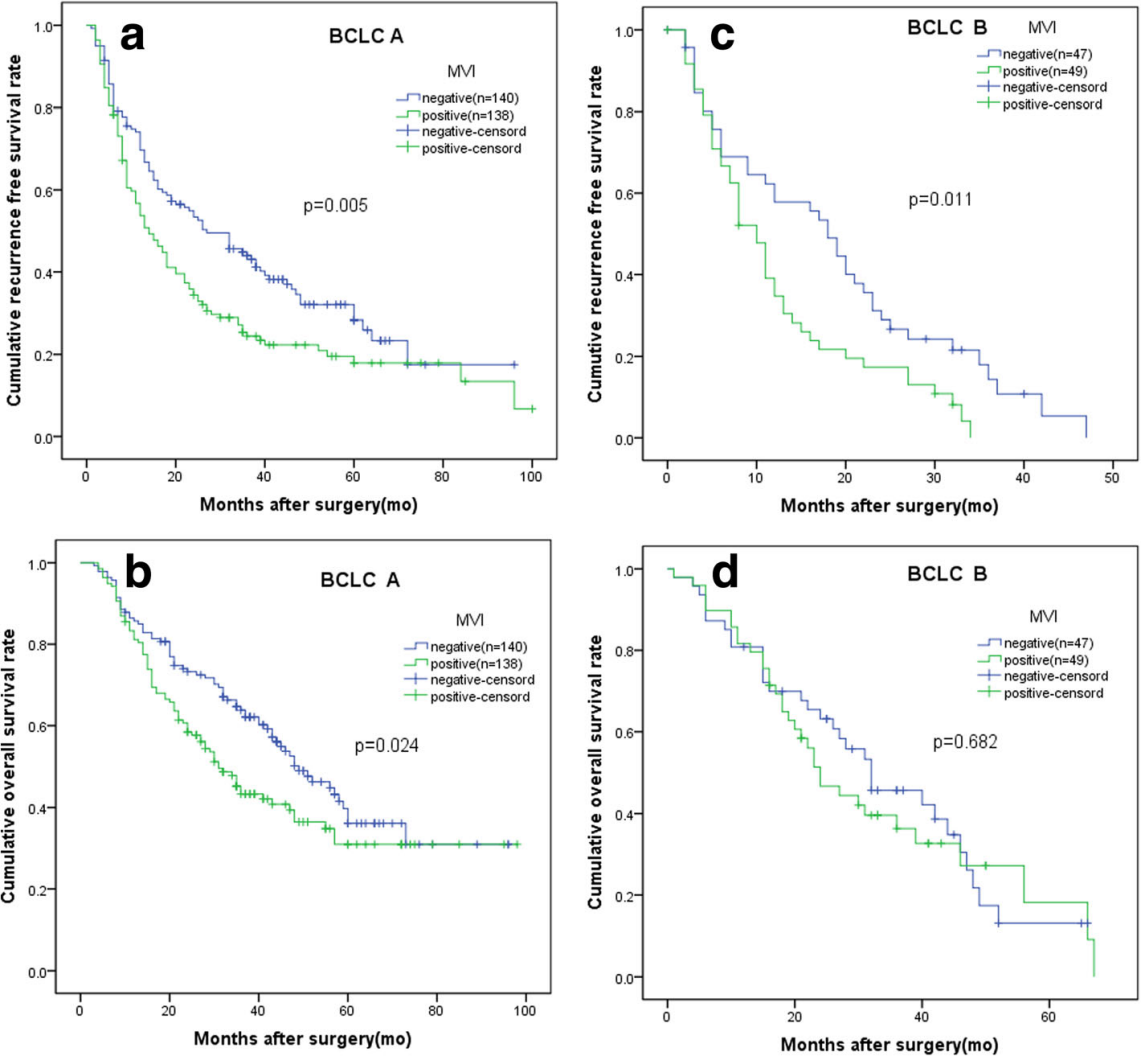
Comparison of patients with or without MVI stratified by BCLC classification after PSM. **a** the recurrence free survival (RFS) rates in patients with BCLC stage A HCCs (*P* = 0.005). **b** The overall survival (OS) rates in patients with BCLC stage A HCCs (*P* = 0.024). **c** the RFS rates in patients with BCLC stage B HCCs (*P* = 0.011). **d** The OS rates in patients with BCLC stage B HCCs (*P* = 0.682)

Comparison of RFS and OS stratified by BCLC classification between the 2 groups in all patients and in the propensity model is presented in Figs. 3 and 4. In BCLC A and B HCCs, MVI-negative group had better RFS than MVI-positive group. After PSM, the baseline characteristics were comparable. Consistently, the RFS rate was statistically significant just in BCLC A and B HCCs.

As to OS, in BCLC A HCCs, before PSM, the 1-, 3- and 5-year OS rates of the MVI-negative group were better than that of the MVI-positive group, with 92.5% vs. 79.9%, 73.9% vs. 43.2%, and 54.6%vs. 31.5%, respectively(*P* < 0.001). After PSM, the OS rates of the MVI-negative group were also better than that of the MVI-positive group, with 85.7% vs. 81.1%, 63.8% vs. 43.3%, and 36.1%vs.30.9%, respectively, for the 1-, 3- and 5-year OS rates (*P* = 0.024).

In BCLC B HCCs, before PSM, the OS rates of the MVI-negative group were better than that of the MVI-positive group, with 86.9% vs. 70.0%, 53.5% vs. 29.6%, and 28.4%vs.14.8%, respectively, for the 1-, 3- and 5-year OS rates (*P* < 0.001). However, after PSM, the OS rates of the MVI-negative group were comparable to that of the MVI-positive group, with 80.9% vs. 81.6%, 45.7% vs. 36.3%, and 13.1%vs. 18.2%, respectively, for the 1-, 3- and 5-year OS rates (*P* = 0.682).

## Discussion

The BCLC staging system is widely used to classify HCC stages in clinical practice because it links separate prognostic groups to various treatment recommendations [2]. Surgical treatment for HCC provides not only a hope for cure but also sufficient pathological information. Major vascular invasion is incorporated into BCLC staging system and classified as BCLC stage C. MVI has been validated as predictor of prognosis of HCC. However the BCLC staging system does not reflect the role of MVI, possibly because of undetectable characteristic of MVI. Surgical liver resection for HCC has evolved into a safe procedure with low surgical morbidity and mortality rates due to improved surgical perioperative care and surgical techniques [19]. MVI can be detected not only through resected specimens but also through noninvasive radiogenomic biomarker that can be evaluated by contrast-enhanced computed tomography (CECT) [14]. The role of MVI should be elucidated in different stage HCCs. HCC is characterized by a tendency for vascular invasion with 37.7% of patients having MVI and 14.8% patients having major vascular invasion in this study. MVI was strongly associated with more tumor numbers, larger tumor size major vascular invasion and poor histological grade. This was in consistent with previous studies showing that MVI could be predicted by tumor size, tumor number and so on [12, 20]. In our study, the MVI-positive had higher rate of intra-and extra-hepatic recurrence compared to MVI-negative patients. The presence of MVI was significantly associated with high systemic inflammation levels, such as PLR, NLR and platelet levels. These systemic inflammatory markers (NLR and PLR) might negative impact the prognosis of cancer [21, 22]. Based on multivariate analysis of OS, tumor size, tumor number and NLR were associated with unfavorable long-term outcomes in MVI-negative HCC. These factors like NLR had been identified by many previous studies. [21] However, in MVI-positive group, more factors such as surgery type, satellite lesions tumor size and serum ALT level were correlated with a decreased overall survival rate. Being consistent with previous studies, these factors have detrimental effects on prognosis [23, 24]. Extended liver resection was associated with prognosis. This is possibly due to its reflection of heavy tumor burden. Another reason might be that extreme liver surgery carries a high rate of morbidity and mortality [25].

Before PSM, patients with MVI had poorer long-term survival than those without MVI (5-year survival rate: 50.0% vs 27.3%). When stratified by BCLC stages, MVI had distinctive power in BCLC stage A and B HCC. However, in the propensity model, after adjusting the confounding factors and reaching comparable baseline characteristics, we confirmed that MVI in HCC patients who underwent hepatectomy was one of the most powerful predictors of overall survival for stage A HCC. Early stages of HCC had higher rate of surgical cure with small tumor burden.. Patients with MVI easily suffered from HCC recurrence. Although MVI is not uncommon in early HCC, it becomes the most important risk factor associated with the prognosis [26]. Even some author suggested that patients with MVI after resection should be recommended for salvage transplantation due to the high rate of recurrence [27]. Unfortunately, the current BCLC staging system does not adopt this high risk factor to stratify subgroups and obtain desirable treatment in early stage HCCs. Postoperative therapy such as TACE may be beneficial for HCC patients with MVI after resection [28].

Interestingly, according to our results, there was a dramatic change in BCLC stage B HCCs with regard to OS before and after PSM. After eliminating the confounding factors, the prognosis was comparable between both groups. As we all know, BCLC stage B HCCs is characterized by multiple tumors and large tumor size with a high proportion of satellite lesions (29.0% in our study) and MVI (41.4%). Even in the same stage, the tumor burden varied greatly. MVI is commonly associated with tumor burden. Before PSM, MVI could predict the prognosis possibly because patients with MVI had more advanced stage HCCs. After the status of tumor reached comparable, MVI had insufficient power to distinguish the OS rate. As a reflection of negative tumor characteristic, MVI was associated with HCC recurrence. The fact that it leads to a high rate of HCC recurrence was consistent with previous studies [29, 30].

In conclusion, based on our propensity model, we proposed that MVI could independently predict the OS in BCLC stage A HCCs. For BCLC stage B HCCs, compared with MVI negative group, MVI positive group had poor prognosis because of more advanced stage tumors.

### Limitations

The strength of the current study lies in the relatively large sample size. All the data were collected from our prospectively maintained database in liver surgery department & liver transplantation cener in the West China Hospital. However, there were several limitations in the current study. First, all patients in this study is from single institution. Therefore, we tried to adopt the PSM method to minimize the selection bias in the study design and analysis. Secondly, the current study included patients predominantly with hepatitis B infection, however, the etiology of HCC in the west is mainly hepatitis C infection.-The conclusion required to be demonstrated in heterogeneous HCC patient population worldwide.. Thirdly, this paper did not study appropriate postoperative treatment strategy for patients with MVI.

## Conclusion

The prognostic value of MVI in different subgroups of BCLC stages differed. It is an independent predictor of prognosis in patients with BCLC stage A. For BCLC stage B HCCs, MVI-positive group had poor prognosis through more advanced HCCs.

## Abbreviation

AASLD: The American Association for the Study of Liver Diseases
AFP: Serum alpha-fetoprotein
ALB: Serum albumin
ALT: Alanine aminotransferase
AST: Aspartate aminotransferase
BCLC: Barcelona Clinical Liver Cancer
HCC: Hepatocelluar carcinoma
MRI: Magnetic resonance imaging
MVI: Microvascular invasion
OS: Overall survival
PSM: Propensity score matching
RFA: Radiofrequency ablation
RFS: Recurrence free survival
SR: Surgical resection
TACE: Transcatheter arterial chemoembolization
TBIL: Total bilirubin
